# Analyzing the Relationship Between Child-to-Parent Violence and Perceived Parental Warmth

**DOI:** 10.3389/fpsyg.2020.590097

**Published:** 2020-11-10

**Authors:** M. Carmen Cano-Lozano, F. Javier Rodríguez-Díaz, Samuel P. León, Lourdes Contreras

**Affiliations:** ^1^Department of Psychology, University of Jaén, Jaén, Spain; ^2^Department of Psychology, University of Oviedo, Oviedo, Spain; ^3^Department of Education, University of Jaén, Jaén, Spain

**Keywords:** anger, child-to-parent violence, drug use, hostile attribution, instrumental reasons, peer group, perceived parental warmth, reactive reasons

## Abstract

The relationship between child-to-parent violence (CPV) and the perceived parental warmth dimension has been well established. However, it is necessary to further investigate the nature of this relationship considering the involvement of other variables. The objective of this study was to analyze the role of cognitive (hostile attribution), emotional (anger), and social variables (deviant peer group and drug use) in the relationship between the perceived parental warmth dimension (warmth-communication and criticism-rejection) and CPV motivated by reactive or instrumental reasons. The community sample consisted of 1,599 Spanish adolescents (54.8% girls) between the ages of 12 and 18 years (*M*_*age*_ = 14.6, *SD* = 1.6 years) from different secondary schools in Jaén (75.3%) and Oviedo (24.7%) (Spain). Each participant completed the Child-to-Parent Violence Questionnaire (CPV-Q), the Warmth Scale (WS), adolescents’ version, the Social Information Processing (SIP) in Child-to-parent Conflicts Questionnaire and Deviant Peers and Drug Use Questionnaires. The results indicate that perceived parental warmth is negatively correlated with hostile attribution, adolescent anger, relationship with a deviant peer group, while perceived parental criticism is positively linked to these variables. Likewise, hostile attribution and adolescent anger are positively linked to reactive CPV. Relationship with a deviant peer group is associated with drug use, which also predicts both reactive and instrumental CPV. In sum, a lack of perceived parental warmth has important repercussions in the form of the psychological and social maladjustment of children, which in turn is differentially correlated with reactive or instrumental CPV. Thus, prevention and intervention programs for CPV should consider, on the one hand, working with parents on parental practices that incorporate parental warmth as a fundamental element and, on the other hand, working with children on cognitive, emotional, and social aspects, taking into account the different motivations for this type of violence.

## Introduction

Child-to-parent violence (CPV) has grown dramatically in the last decade, leading to an increase in research on this topic in different countries (e.g., Beckman et al., 2017 in Germany; Contreras and Cano-Lozano, 2015, 2016 in Spain; Margolin and Baucom, 2014 in the United States; Pagani et al., 2009 in Canada; and Simmons et al., 2018 in Australia). This type of family violence has been defined as “any act of a child that is intended to cause physical, psychological, or financial damage to gain power and control over a parent” (Cottrell, 2001, p. 3). More recently, other authors note that this type of violent behavior is also aimed to dominate parents (Howard and Rottem, 2008; Molla-Esparza and Aroca-Montolío, 2018).

There are a wide variety of behaviors that reflect different types of CPV. Following Cottrell (2001), psychological violence includes, for example, intimidations or threats and also verbal behaviors such as insulting or shouting. Physical violence refers to acts such as punching, pushing or kicking. Financial violence includes behaviors such as stealing money, destroying the home or incurring debts the parents must cover. The control, power and domination over parents is reflected in such behaviors as making unrealistic demands on parents (for example, insisting they drop what they’re doing to comply with the child’s demands) or controlling the running of the household. These types of abuse can occur at the same time, and in fact, they overlap to a certain extent (Cottrell, 2001), resulting in an escalation of violence from psychological abuse to more severe form of violence such as physical abuse (Cottrell, 2001; Eckstein, 2004). In addition, in line with what has been indicated for other types of violence that manifest in other contexts, different authors have pointed out that CPV can be reactive or instrumental (Calvete et al., 2015; Calvete and Orue, 2016; Contreras et al., 2019, 2020). Reactive violence is characterized by anger (Poulin and Boivin, 2000) and hostile attributions (Orobio de Castro et al., 2002; Arsenio et al., 2009) and is a response to a previous provocation, real or perceived (Crick and Dodge, 1996). Instrumental violence refers to the use of aggression to obtain what one wants to get something (Crick and Dodge, 1996).

The prevalence rates of CPV, although quite different depending on the characteristics of the study, are very high, which shows the magnitude of the problem. Studies from Canada and the United States, applying as the CPV criterion the occurrence of violent behavior on at least one occasion, have found percentages of verbal violence toward mothers between 19 and 64% and toward fathers between 8 and 56%. The percentage of mothers who have experienced physical violence ranges between 8 and 13.8%, and that of fathers is 6–11% (Pagani et al., 2004, 2009; Margolin and Baucom, 2014). For financial violence, the percentages are 22% for mothers and 11% for fathers (Margolin and Baucom, 2014). In Spain, the percentages for psychological violence are 90.6–92.2% for mothers and 79.5–86.5% for fathers, whereas the percentages for physical violence are 6.4–19.1% toward mothers and 5.4–16.6% toward fathers (Calvete and Orue, 2016; Calvete et al., 2017; Rico et al., 2017). For financial violence, the percentages are 26.9% for mothers and 23.7% for fathers (Rico et al., 2017).

In recent years, research on this phenomenon has been extensive, generating abundant information about the relationship between various individual, family, and social variables and the development and maintenance of CPV. In this sense, the study of variables related to the family environment has aroused great interest because this is the context in which this type of violence takes place (Ibabe, 2016; Gallego et al., 2019). More specifically, in the analysis of family dynamics, it has been common to resort to the study of parenting styles. Maccoby and Martin (1983) redefined the initial proposal of three parenting styles (democratic, authoritarian, and permissive) of Baumrind (1971) into to two dimensions: (a) responsiveness, which refers to affective, warmth, acceptance, and support, and (b) demandingness, which refers to the use of control and supervision. From the combination of these two dimensions, four parenting styles emerge: authoritarian, authoritative, permissive-indulgent and neglectful.

The relationship between parenting style and CPV is complex. Some studies have found a relationship between CPV and the authoritarian style in community samples (Ibabe et al., 2013; Suárez-Relinque et al., 2019) and between CPV and a permissive and neglectful style in both community samples (Gámez-Guadix et al., 2012; Ibabe et al., 2013) and forensic samples (Castañeda et al., 2012; Contreras and Cano-Lozano, 2014). However, other studies with community samples have not found a relationship between the permissive style and CPV (Calvete et al., 2015; Suárez-Relinque et al., 2019). Considering this scenario, it has been considered more useful at the empirical level to focus on specific parental dimensions or practices.

Studies that analyze parental dimensions separately agree that the responsiveness dimension makes the difference in CPV. Specifically, parental warmth is a protective factor against physical CPV from adolescent girls (Beckman et al., 2017). In addition, both studies with adolescents and young people have found that the absence of parental warmth is fundamental in the development of CPV (Gámez-Guadix et al., 2012; Calvete et al., 2015). Other studies highlight the importance of the maternal figure in this dimension. For example, Ibabe et al. (2013) found that CPV was associated with emotional rejection by the mother. In the forensic field, Contreras and Cano-Lozano (2014) identified that what differentiated juveniles charged with CPV offenses from other juvenile offenders was precisely the parental warmth dimension. Specifically, juveniles charged with CPV offenses perceived less warmth and more criticism, especially from their mothers, than juveniles charged with other types of crimes and non-offending minors. More recently, Zhang et al. (2019) found that maternal emotional warmth is associated with fewer behaviors of contempt and rebellion toward mothers by adolescents and that maternal rejection is related to more rebellion behaviors toward the mother.

However, the lack of parental warmth as a risk factor does not explain by itself how this leads adolescents to be violent toward their parents. The effects of the lack of parental warmth on the problematic behaviors of the children may be influenced by other variables. The interpersonal acceptance-rejection (IPAR) theory is an evidence-based theory that attempts to explain and predict the main antecedents, consequents, and correlates of parental acceptance/rejection (Rohner et al., 2012). Parental acceptance refers to warmth, affection, support, or simply the love of parents toward their children. Parental rejection, in turn, refers to the absence or withdrawal of some of these aspects. According to IPAR theory, parental rejection can be expressed by: (1) coldness/lack of affection; (2) hostility/aggression; (3) indifference/neglect; and (4) undifferentiated rejection. According to the theory, there is a biological need for acceptance from the most significant people. Thus, children need to be accepted by their parents, that is, they need to feel parental warmth, affection or support. More specifically, individuals who perceive parental rejection are likely to develop (1) anger, hostility/aggression, (2) dependence or defensive independence, (3) negative self-esteem, (4) negative self-adequacy, (5) emotional instability, (6) lack of emotional response, and (7) a negative worldview (Rohner, 1999). People who feel rejected are likely to develop a negative worldview (Rohner, 1999). This has significant negative effects on the psychological adjustment of children and on their behavior and relationships with others.

The relationship between perceived parental rejection and the psychological maladjustment of children has been identified in many studies (e.g., Khaleque and Rohner, 2002, 2012; Khaleque, 2013, 2015) and statistically confirmed in meta-analytic studies (e.g., Khaleque, 2013, 2017). In a meta-analysis that included 30 studies from 16 countries, Khaleque (2013) found that perceived maternal and paternal warmth/affection were positively related with psychological adjustment, independence, positive self-esteem, positive self-adequacy, emotional responsiveness, emotional stability, and positive worldview and negatively related with children’s self-reports about hostility/aggression. A more recent meta-analysis by Khaleque (2017) found that both perceived maternal and paternal hostility and aggression were positively related with the psychological maladjustment of children and the seven negative personality dispositions. The results also indicate that the relationships are slightly but significantly stronger in mothers than in fathers.

In early childhood, the regulation of emotions and behaviors depends largely on parental support (Eisenberg et al., 1998; Morris et al., 2007). Some researchers (Gottman et al., 1997; Eisenberg et al., 1998) have suggested that one reason for the association between parental warmth/positive expressivity and child externalization problems is its effects on emotional regulation in children. According to this view, warm, positive parents contribute to the regulation of their children. Along these lines, the emotional socialization practices of parents promote self-regulation skills in children and reduce the risk of external symptoms (e.g., Eisenberg et al., 2005; Valiente et al., 2007). Likewise, some children who experience negative parental affection may feel rejected by their parents and this can promote the development of internalizing symptoms. Moreover, children can also develop externalizing problems by imitating the negative emotional expression of the parents (Stocker et al., 2007). In short, perceived parental rejection is one of the main causes of behavioral problems in childhood and adolescence, and it could have these effects through cognitive and emotional variables.

In the context of CPV, few studies have analyzed cognitive and emotional variables, although these variable types have recently aroused the interest of different researchers. Regarding the cognitive variables, hostile attribution in adolescents is prominent in the development of CPV (Calvete et al., 2015; Rosado et al., 2017). Contreras and Cano-Lozano (2015, 2016), in their studies of forensic samples, indicated that minors who had committed CPV offenses presented a more hostile perception of their parents and their home in general than other juvenile offenders and non-offenders. The literature on general violent behavior indicates that hostile attribution is linked to reactive violence (Orobio de Castro et al., 2002; Arsenio et al., 2009), although in a previous study on CPV, this specific relationship with reactive violence was not found (Contreras et al., 2020), so it is necessary to continue investigating this issue. Regarding emotional variables, adolescents who assault their parents often have emotional difficulties, specifically in controlling (Beckman et al., 2017), identifying, and expressing their emotions (Martínez-Ferrer et al., 2018). One of the most relevant emotional variables is anger, which makes them more likely to behave aggressively in general (e.g., Fives et al., 2011). In this context, anger is a fundamental variable in the development of CPV (Calvete et al., 2015; Loinaz and de Sousa, 2019), and this variable predicts CPV toward the mother (Orue et al., 2019). These results are confirmed in samples of young people aged 18–25 years, with anger being a predictor of CPV toward both parents (Simmons et al., 2020). Other studies have delved further into this variable, indicating that anger predicts reactive CPV toward both the father and the mother (Contreras et al., 2020).

The perceived parental warmth dimension has also been related to problematic behavior in adolescents through the roles of other social variables, such as relationship with a deviant peer group and drug use. Low maternal support has been indirectly related to participation in criminal activities through the child’s affiliation with deviant peers (Deutsch et al., 2012). Trudeau et al. (2012) found that parenting that includes affection, discipline, standard setting, and monitoring indirectly predicts, through deviant peers, externalizing problems, including violent and aggressive behavior. Van Ryzin and Dishion (2013) showed that coercive family interactions led to coercive relationships with peers and, consequently, to violent behavior in early adulthood. In contrast, although other studies found that the effects of parental knowledge on different types of problematic behaviors were mediated by the child’s affiliation with deviant peers, they did not find significant effects of parental support, parental control, and parental solicitation (Cutrín et al., 2019). In turn, monitoring and quality in family relationships has been correlated with smoking and drinking through deviant peer groups (Van Ryzin et al., 2012). More specifically, parenting is related to externalizing behavior problems through deviant peers, and parenting is related to drug use through peers who use drugs (Cox et al., 2017).

In the field of CPV, research on these social variables is much scarcer, but in general, studies conducted on both community samples and clinical and forensic samples reveal that adolescents who assault their parents tend to relate with deviant peer groups (Kennedy et al., 2010; Calvete et al., 2011; Castañeda et al., 2012; Del Moral et al., 2015; Loinaz and de Sousa, 2019). As suggested by Cottrell and Monk (2004), the peer group constitutes a behavioral model in which violence is used to obtain power and control over others so that adolescents learn these violent behaviors and use them in their relationships with their parents. Regarding the study of drug use in the field of CPV, numerous studies on adolescents show that drug use is positively associated with this type of violent behavior (Calvete et al., 2011; Ibabe et al., 2013; Beckman et al., 2017; Rico et al., 2017; Rosado et al., 2017). In this sense, some researchers point out that drug use increases the risk of verbal aggression toward the father and mother by approximately 50–60% (Pagani et al., 2004, 2009). However, as noted by Simmons et al. (2018), in community samples, the effect sizes are small, and in forensic samples, the use rates are similar to those of offenders in general (Contreras and Cano-Lozano, 2015), suggesting that substance use may be part of an underlying pattern of antisocial behavior rather than a specific causal factor in child-to-parent abuse (Simmons et al., 2018). In any case, what seems to be true is that drug use clearly contributes to the emergence of conflicts between parents and children (Contreras and Cano-Lozano, 2015; Armstrong et al., 2018) and that this can occur in different ways because the relationship is complex. In turn, reactive violent behaviors (characterized by an intense emotional response) occur under the influence of drugs due to the verbal and behavioral disinhibition engendered by drug use (Goldstein, 1995). In the context of CPV, frequent substance use can facilitate verbal disinhibition in confrontations with parents, increasing the risk of violent verbal behavior (Pagani et al., 2004) that can escalate to physical aggression (Pagani et al., 2009). In fact, Contreras and Cano-Lozano (2015) observed in forensic sample that 46.7% of minors charged with offenses of abuse toward their parents admitted that the aggressions had taken place under the influence of drugs. In turn, there are also instrumental or functional violent behaviors exercised mainly to obtain money for drugs (Goldstein, 1995). Recent studies indicate that getting more money from parents is one of the reasons for CPV (Calvete and Orue, 2016; Contreras et al., 2019, 2020).

The literature also reveals a close relationship between a deviant peer group and drug use during adolescence (e.g., Fergusson et al., 2002; Duan et al., 2009; Kendler et al., 2014). Regarding CPV, in Spain, it has been observed recently that a deviant peer group predicts drug use, which in turn is linked to violent behavior toward parents (Del Hoyo-Bilbao et al., 2020), i.e., there is an indirect effect of the deviant peer group on CPV through drug use. At the same time, these authors found that affiliation with a deviant peer group was influenced by family variables such as a lack of parental support or parental inefficiency.

### Current Study

The previous literature shows the relationship between CPV and the perceived parental warmth dimension, but it is necessary to further investigate this relationship given the complexity of the topic. It is likely that the effects of perceived lack of parental warmth on CPV occur through other variables. In other research fields, numerous studies have identified a relationship between perceived parental rejection and the psychological maladjustment of children, but no study has analyzed it specifically in relation to CPV. In addition, it would be of great interest to identify the reasons that motivate CPV according to the detected effects. Thus, the purpose of this study is to further investigate the relationship between the perceived parental warmth dimension and CPV through other variables, including cognitive, emotional and social variables. More specifically, our objective is to analyze the role of cognitive (hostile attribution), emotional (anger), and social variables (deviant peer group and drug use) in the relationship between the perceived parental warmth dimension (warmth-communication and criticism-rejection) and CPV motivated by reactive or instrumental reasons. The hypotheses of this study were as follows: (1) Warmth-communication is negatively correlated with anger, hostile attribution, and relationship with a deviant peer group (Trudeau et al., 2012; Khaleque, 2013), while criticism-rejection is positively correlated with these variables (Khaleque, 2017; Van Ryzin and Dishion (2013). (2) Hostile attribution (Orobio de Castro et al., 2002; Arsenio et al., 2009) and anger (Poulin and Boivin, 2000; Contreras et al., 2020) are positively correlated with CPV motivated by reactive reasons. (3) Relationship with a deviant peer group is positively correlated with drug use (Del Hoyo-Bilbao et al., 2020), which in turn is positively correlated with CPV motivated both by reactive reasons (Pagani et al., 2004; Contreras and Cano-Lozano, 2015) and instrumental reasons (Calvete and Orue, 2016; Contreras et al., 2019, 2020).

## Materials and Methods

### Sample

The sample was made up of 1,599 Spanish adolescents (54.8% girls) aged between 12 and 18 years (*M*_*age*_ = 14.6, *SD* = 1.6 years) from a community population and they were recruited from eight public and private secondary schools in Jaén (75.3%) and Oviedo (24.7%) (Spain). Regarding marital status, most of the parents were married (83.4%).

Previously, the minimal sample size was calculated at 95% confidence level, with a 5% confidence interval at 80% of statistical power. The estimated minimum sample size was 385. According to Hair et al. (2010), the general rule to calculate the minimum sample size for factor treatment in a survey is to have a minimum of 5 observations per variable (5:1). In the current study, the scales consisted of 138 items, so the minimum for the factorial treatment would be 690.

### Instruments

The information on the validity and reliability of all assessment instruments in this study is described in the “Results” section.

The Child-to-Parent Violence Questionnaire (CPV-Q) (Contreras et al., 2019). The CPV-Q consists of 14 parallel items (for the father and for the mother) that measure psychological (four items), physical (three items), and financial violence (three items), together with behaviors of control and dominion over their parents (four items). The CPV-Q asks the adolescents to indicate the frequency of the behaviors against their parents in the past year using a 4-points scale: 0 (never), 1 (rarely = it has occurred once), 2 (sometimes = 2–3 times), 3 (many times = 4–5 times), and 4 (very often = more than 6 times). It also includes a scale with 8 items on the reasons for the aggressions, 3 items referring reactive reasons (RR) and 5 items to instrumental reasons (RR), each answered a 3-points scale: 0 (never), 1 (sometimes), 2 (almost always), and 3 (always). Higher scores indicate more CPV and more frequency of RR an IR.

The Warmth Scale (WS), adolescents’ version (Fuentes et al., 1999). The WS is made up of 20 items, divided into two factors: (a) Warmth-communication and (b) Criticism-rejection by parents toward their children. Each factor consists of 10 items rated on a scale ranging from 1 (never) to 5 (always). Higher scores indicate more warmth-communication and more criticism-rejection.

The Social Information Processing (SIP) in Child-to-parent Conflicts Questionnaire (Calvete et al., 2015). The anger and hostile attribution scales were used for this study. Adolescents were asked to imagine three scenes of different conflicts with their parents, and they had to respond to each item on a 5-point response scale ranging from 0 (not at all) to 4 (to a great extent): (a) hostile attribution, which included the attribution of negative intentions and positive emotions in parents (2 items per scene, 6 items in total); (b) anger (1 item per scene, 3 items in total). Higher scores indicate more anger and hostile attributions.

Deviant Peers Questionnaire. This instrument was designed *ad hoc* for this study. It has a total of four items with which adolescents are asked to indicate if their friends have been involved in criminal activities, show violent behavior, cut school, and/or use drugs. The response scale is 1 (none of them) to 4 (all). Higher scores indicate more frequency of relationship with deviant peer groups.

Drug Use Questionnaire. This instrument was designed *ad hoc* for this study. Adolescents were asked to indicate how often they have used different drugs (tobacco, alcohol, marijuana, hashish, cocaine, speed, ecstasy) in the last year, on a scale of 1 (never) to 5 (daily). Higher scores indicate more frequency of drug use.

### Procedure and Design

First, the favorable report of the Ethics Committee of the University of Jaén (Spain) to conduct this study was obtained (Ref. CEIH 270215-1). Then, authorizations by the Public Administration in Education and the secondary schools’ directors were also obtained. The secondary schools were previously selected by the Provincial Delegations of Education according to their representativeness. Eight secondary schools were invited to participate and they were given detailed information of the objectives of the research. The parents’ informed consent for us to assess their children and the adolescent’s informed consent were also requested. Those schools that confirmed their availability and willingness to take part in the research provided the informed consent in paper to both parents and children. Adolescents received the same information as their parents and they participated in the study once they have signed the informed consent. In the case of adolescents under 18 years, they participated in the assessment only if they had given their informed consent and that of their parents. Each participant received an identification code and no incentive was offered in exchange for participation. The questionnaires in paper were administered in a group setting in their classrooms. The evaluation time was approximately one hour. Three evaluators from the research group, who were specifically trained for this protocol, conducted the evaluations. Data collection was conducted during 2017 and 2018. The inclusion criteria were to be aged between 12 and 18 years old and to have the informed consent from parents to participate in the study. Participants under 12 years and above 18 were excluded.

This is a survey descriptive study using cross-sectional research design (Montero and León, 2007).

### Data Analysis

All analyses were performed in R software. The *p*-value for all tests was set at 0.05. Missing values were computed by multiple imputation using the R package MICE (Buuren and Groothuis-Oudshoorn, 2011). Before factorial analysis of the data, data were screened to analyze the distributions and test statistical assumptions before analysis. To test the assumptions, a regression was created with our data and a group of random data, and the distribution of the residuals was analyzed. If there was any anomaly in the distribution of the residuals, this would be due to the distribution of our data. Confirmatory factor analysis (CFA) of the questionnaires used in the study and structural equation modeling (SEM) were performed with the lavaan R package (Rosseel, 2012). The diagonal weighted least squares (DWLS) estimator was used for CFA due to the non-normal multivariate distribution of the data. The fit indices used in CFA were Comparative Fit Index (CFI), Tucker-Lewis Index (TIF), Standardized Root Mean Square Residual (SRMS), and Root Mean Square Error of Approximation (RMSEA) with 90% of Confident interval. The latent variables that constituted the different elements in the SEM model were computed by multiplying the observed variables that comprised them. For SEM, maximum likelihood estimation with robust standard errors and the Satorra-Bentler scaled test (Maximum Likelihood Method, MLM) were used. Cronbach’s alpha and McDonald ω were used to assess reliability of each subscale.

## Results

Of all the possible answers given by the participants on the different questionnaires, only 2.75% were missing. The multivariate normality of the data was analyzed using the Mardian test, and the results showed that the data did not have a multivariate normal distribution (Zkurtosis 811.98, *p* < 0.01). No item showed multicollinearity (*r* > 0.90) or singularity (*r* > 0.95). Data screening showed that the data did not violate the assumption of linearity, homogeneity, or homoscedasticity (the residuals of the false regression were mostly distributed between −2 and +2).

### CFA of the Questionnaires

Before analyzing the proposed SEM model, the validity and reliability of the questionnaires used in the present study were calculated (see Table 1). To do this, a CFA of all the questionnaires was performed. The results showed that the goodness of fit determined by the CFA was between good and excellent for each questionnaire (Hair et al., 2010). Below are the results for each of them:

**TABLE 1:** Model fit parameter estimates by subscale.

								RMSEA 90% CI	
Scale	χ*^2^*	*Df*	*P*	CFI	TIF	SRMS	RMSEA	Lower	Upper
CPV-F	80.474	73	0.257	0.996	0.995	0.066	0.008	0.000	0.017
CPV-M	84.204	73	0.174	0.995	0.994	0.057	0.010	0.000	0.018
Reasons	82.111	19	< 0.001	0.960	0.941	0.059	0.046	0.036	0.052
W-F	503.235	169	< 0.001	0.991	0.989	0.050	0.035	0.032	0.039
W-M	381.024	169	< 0.001	0.990	0.988	0.045	0.028	0.024	0.032
SIP	175.659	26	< 0.001	0.965	0.951	0.066	0.060	0.052	0.023
Deviant peers	16.456	2	< 0.001	0.975	0.925	0.040	0.067	0.040	0.099
Drugs use	16.771	9	0.052	0.988	0.980	0.140	0.023	0.000	0.040

*CPV-F, Child-to-Parent Violence Questionnaire - Father; CPV-M, Child-to-Parent Violence Questionnaire - Mother; W-F, Warmth - Father; W-M, Warmth - Mother; SIP, Social Information Processing Scale.*

#### CPV-Q-Father

The CFA showed an excellent fit (χ^2^73 = 80.474, *p* = 0.257; see Table 1 for more details), with comparative fit index (CFI) = 0.996, Tucker-Lewis index (TLI) = 0.995, standardized root mean squared residual (SRMR) = 0.066, root mean square error of approximation (RMSEA) = 0.008 (RMSEA 90% CI [0.000,0.017]), and reliability indices of α = 0.820 and ω = 0.837.

#### CPV-Q-Mother

The CFA showed an excellent fit (χ^2^73 = 84.204, *p* = 0.174; see Table 1 for more details), with CFI = 0.995, TLI = 0.994, SRMR = 0.057, RMSEA = 0.010 (RMSEA 90% CI [0.000,0.018]), and reliability indices of α = 0.822 and ω = 0.843.

#### Questionnaire on Reasons for CPV

The CFA showed a good fit (χ^2^19 = 82.111, *p* < 0.001; see Table 1 for more details), with CFI = 0.960, TLI = 0.941, SRMR = 0.059, RMSEA = 0.046 (RMSEA 90% CI [0.036,0.052]), and reliability indices of α = 0.718 and ω = 0.747 for the overall scale and α = 0.668 and ω = 0.618 for RR and α = 0.704 and ω = 0.703 for IR.

#### Warmth Scale-Father

The CFA showed an excellent fit (χ^2^169 = 503.235, *p* < 0.001; see Table 1 for more details), with CFI = 0.991, TLI = 0.989, SRMR = 0.050, RMSEA = 0.035 (RMSEA 90% CI [0.032,0.039]), and reliability indices of α = 0.500 and ω = 0.714 for the overall scale and α = 0.919, ω = 0.920 for the Warmth-Communication dimension, and α = 0.887 and ω = 0.889 for the Criticism-Rejection dimension.

#### Warmth Scale-Mother

The CFA showed an excellent fit (χ^2^169 = 381.024, *p* < 0.001; see Table 1 for more details), with CFI = 0.990, TLI = 0.988, SRMR = 0.045, RMSEA = 0.028 (RMSEA 90% CI [0.024,0.032]), and reliability indices of α = 0.417 and ω = 0.634 for the overall scale, α = 0.887 and ω = 0.889 for the Warmth-Communication dimension, and α = 0.843 and ω = 0.842 for the Criticism-Rejection dimension.

#### Social Information Processing in Child-to-parent Conflicts Questionnaire, Hostile Attribution and Anger Subscales

The CFA showed a good fit (χ^2^26 = 175.659, *p* < 0.001; see Table 1 for more details), with CFI = 0.965, TLI = 0.951, SRMR = 0.066, RMSEA = 0.060 (RMSEA 90% CI [0.052,0.023]), and reliability indices of α = 0.800 and ω = 0.811 for the overall scale, α = 0.720 and ω = 0.712 for hostile attribution, and α = 0.745 and ω = 0.745 for anger.

#### Deviant Peers Questionnaire (*ad hoc*)

The CFA showed a good fit (χ^2^2 = 16.456, *p* < 0.001; see Table 1 for more details), with CFI = 0.975, TLI = 0.925, SRMR = 0.040, RMSEA = 0.067 (RMSEA 90% CI [0.040,0.099]), and reliability indices of α = 0.647 and ω = 0.648.

#### Drug Use Questionnaire (*ad hoc*)

The CFA showed an excellent fit (χ^2^9 = 16.771, *p* = 0.052; see Table 1 for more details), with CFI = 0.988, TLI = 0.980, SRMR = 0.140, RMSEA = 0.023 (RMSEA 90% CI [0.000,0.040]), and reliability indices of α = 0.721 and ω = 0.665.

### Structural Model Approach

**FIGURE 1:**
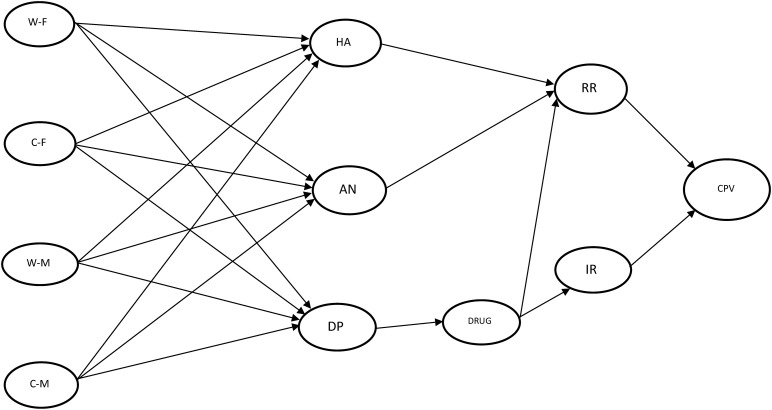
SEM theoretical model for Child-to-Parent Violence (CPV). The circles represent the latent variables, and the arrows indicate the regression between variables. W-F, Warmth-Father; C-F, Criticism-Father; W-M, Warmth-Mother; C-M, Criticism-Mother; HA, Hostile Attribution; AN, Anger; DP, Deviant Peers; RR, Reactive Reasons; IR, Instrumental Reasons.

**FIGURE 2:**
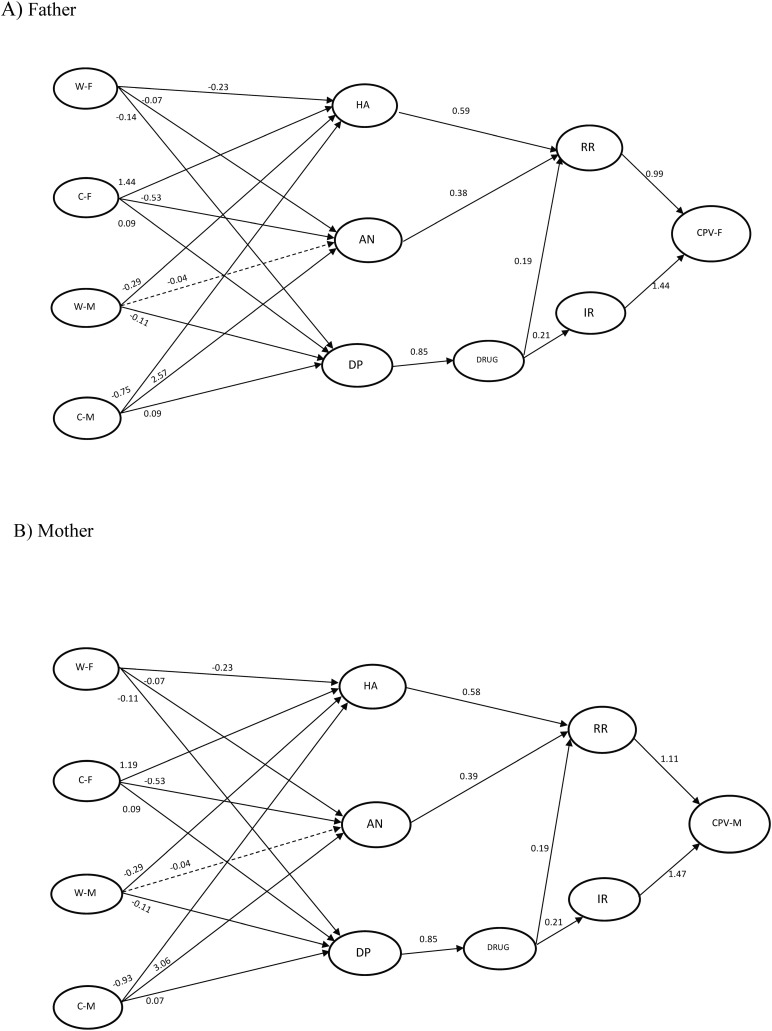
Results of the structural equation models. The circles represent the latent variables, and the arrows indicate the regression between variables. The solid arrows represent significant relationships whereas the dotted arrows indicate non-significant relationships. The numbers indicate the standardized value of the factor load of each variable in the model. W-M, Warmth-Mother; C-M, Criticism-Mother; W-F, Warmth-Father; C-F, Criticism-Father; HA, Hostile Attribution; AN, Anger; DP, Deviant Peers; RR, Reactive Reasons; IR, Instrumental Reasons; CPV-F, Child-to-Parent Violence-Father; CPV-M, Child-to-Parent Violence-Mother. The model for fathers is presented in the upper panel **A**, and the model for mothers is presented on the panel **B**.

The conceptual model proposed to understand the relationships between the factors involved in perceived parental warmth and reactive and instrumental CPV is presented in Figure 1. This model will be applied to CPV toward fathers and mothers. The results of the SEM analysis showed an excellent fit for the model applied to fathers (χ^2^21 = 179.814, *p* < 0.001, CFI = 0.965, TLI = 0.908, SRMR = 0.065, RMSEA = 0.069 (RMSEA 90% CI [0.061,0.077]). Akaike’s information criterion (AIC) = 37,207.645, and the Bayesian information criterion (BIC) = 37,444.266. The SEM analysis also showed an excellent fit for the model applied to mothers (χ^2^21 = 247.525, *p* < 0.001, CFI = 0.951, TLI = 0.873, SRMR = 0.073, RMSEA = 0.082 (RMSEA 90% CI [0.074,0.090]), with AIC = 37,182.305 and BIC = 37,418.927. Tables 2, 3 show in detail the results of the SEM analysis for each of the models. Figure 2 represents the results of the analysis of the models proposed in the case of fathers (Figure 2A) and in the case of mothers (Figure 2B). In both models all the relationships (except between warmth-mother and anger) were significant. Both models (Father and Mother) show similar factor loadings between the relationships of the different components of the model.

**TABLE 2:** Regression factors from structural equation modeling for father.

Father	Estimate	SE	*Z*	*p*	Std. Estimate
**Anger**					
W-M	–0.038	0.028	–1.376	0.169	–0.039
C-M	2.573	0.588	4.373	< 0.001	2.623
W-F	–0.067	0.027	–2.455	0.014	–0.067
C-F	–0.532	0.085	–6.273	< 0.001	–0.549
**HA**					
W-M	–0.291	0.033	–8.910	< 0.001	–0.289
C-M	–0.755	0.267	–2.826	0.005	–0.761
W-F	–0.228	0.031	–7.444	< 0.001	–0.227
C-F	1.441	0.157	9.191	< 0.001	1.471
**Deviant peers**					
W-M	–0.115	0.025	–4.633	< 0.001	–0.115
C-M	0.085	0.029	2.903	0.004	0.086
W-F	–0.143	0.024	–5.927	< 0.001	–0.144
C-F	0.086	0.030	2.888	0.004	0.089
**RR**					
Anger	0.384	0.026	14.782	< 0.001	0.384
HA	0.592	0.056	10.589	< 0.001	0.598
Drug use	0.194	0.028	7.047	< 0.001	0.195
**Drug use**					
Deviant Peers	0.847	0.080	10.561	< 0.001	0.846
**IR**					
Drug use	0.207	0.028	7.415	< 0.001	0.204
**CPV-F**					
IR	0.986	0.080	12.387	< 0.001	0.341
RR	1.439	0.082	17.572	< 0.001	0.488

*W-M, Warmth-Mother; C-M, Criticism-Mother; W-F, Warmth-Father; C-F, Criticism-Father; HA, Hostile Attribution; RR, Reactive Reasons; IR, Instrumental Reasons; CPV-F, Child-to-Parent Violence-Father.*

**TABLE 3:** Regression factors from structural equation modeling for mother.

Mother	Estimate	SE	*Z*	*p*	Std. Estimate
**Anger**					
W-M	–0.038	0.028	–1.388	0.165	–0.039
C-M	3.059	0.637	4.800	< 0.001	3.149
W-F	–0.067	0.027	–2.490	0.013	–0.067
C-F	–0.399	0.068	–5.862	< 0.001	–0.410
**HA**					
W-M	–0.291	0.033	–8.934	< 0.001	–0.288
C-M	–0.929	0.296	–3.144	0.002	–0.942
W-F	–0.228	0.031	–7.458	< 0.001	–0.226
C-F	1.194	0.129	9.265	< 0.001	1.209
**Deviant peers**					
W-M	–0.115	0.025	–4.642	< 0.001	–0.115
C-M	0.072	0.031	2.349	0.019	0.074
W-F	–0.143	0.024	–5.942	< 0.001	–0.144
C-F	0.088	0.029	3.069	0.002	0.090
**RR**					
Anger	0.388	0.027	14.266	< 0.001	0.388
HA	0.584	0.051	11.540	< 0.001	0.593
Drug use	0.194	0.028	6.899	< 0.001	0.195
**Drug use**					
Deviant Peers	0.847	0.079	10.766	< 0.001	0.847
**IR**					
Drug use	0.207	0.029	7.205	< 0.001	0.202
**CPV-M**					
IR	1.105	0.075	14.658	< 0.001	0.409
RR	1.474	0.077	19.159	< 0.001	0.531

*W-M, Warmth-Mother; C-M, Criticism-Mother; W-F, Warmth-Father; C-F, Criticism-Father; HA, Hostile Attribution; RR, Reactive Reasons; IR, Instrumental Reasons; CPV-M, Child-to-Parent Violence-Mother.*

## Discussion

The objective of this study was to further investigate the relationship between perceived parental warmth and CPV. More specifically, it looked into the role of cognitive (hostile attribution), emotional (anger), and social variables (deviant peer group and drug use) in the relationship between perceived parental warmth-communication and criticism-rejection and CPV motivated by reactive and instrumental reasons.

Hypothesis 1 holds that perceived parental warmth-communication is negatively correlated with hostile attribution, anger and a deviant peer group, while perceived parental criticism-rejection is positively correlated with these variables. According to IPAR theory, individuals who perceive parental rejection, manifested by both coldness or lack of affection and hostility of the parents toward the child, are likely to develop various problems, including hostility and anger. Our results partially confirm this hypothesis. Indeed, perceived paternal and maternal warmth were negatively correlated with hostile attribution, and perceived paternal warmth was negatively correlated with anger, but in the case of the mother, this last relationship was not significant. In the case of the criticism-rejection dimension, the results were as expected, except for perceived paternal criticism and anger and perceived maternal criticism and hostile attribution, whose relationship was contrary to the expected. In general, the results agree with various studies that have found a relationship between perceived parental rejection and psychological maladjustment of children in the form of problems of hostility and emotional regulation, among others (Khaleque and Rohner, 2002, 2012; Khaleque, 2013, 2015). However, it is true that some results are unexpected, so this aspect needs to be replicated and further analyze the differences between fathers and mothers. The strongest relationship we observed was between perceived maternal criticism and child anger. This finding agrees with the review conducted by Khaleque (2017), who found that perceived maternal hostility/aggression showed a stronger relationship with psychological maladjustment of children than perceived paternal hostility/aggression. The reason for this result is not clear. A possible explanation is that children spend more time and have stronger relationships with mothers than with fathers. Further research is needed to clarify and explain this result (Khaleque, 2017).

The perceived parental warmth dimension has also been correlated with externalizing problems through the role of the deviant peer group and drug use. In this sense, our data indicate, in line with our expectations, that while perceived paternal and maternal warmth are negatively correlated with having a deviant peer group, perceived paternal and maternal criticism-rejection are positively correlated with having a deviant peer group. Trudeau et al. (2012) also found that lack of parental affection, among other parenting behaviors, predicted violent and aggressive behavior in children through deviant peer association. With respect to perceived parental criticism-rejection, the data are in line with the data of Van Ryzin and Dishion (2013), who found that family coercive interactions led to coercive relationships with peers and thus to violent behavior.

Hypothesis 2 proposed that hostile attribution and anger would be positively correlated with CPV motivated by reactive reasons. The results confirmed this hypothesis in the case of both fathers and mothers. Regarding hostile attribution, different studies on this variable have indicated its importance in the development of CPV (Calvete et al., 2015; Contreras and Cano-Lozano, 2015; Rosado et al., 2017), and this variable is linked to general reactive violence (Orobio de Castro et al., 2002; Arsenio et al., 2009), which is consistent with our results. Anger also predicts this type of aggression toward parents (Calvete et al., 2015; Orue et al., 2019; Simmons et al., 2020). In addition, the literature on general violent behavior has indicated that this variable is specifically linked to reactive violence (Poulin and Boivin, 2000). Our study provides additional evidence on this topic, since anger was positively correlated with CPV toward the father and toward the mother motivated by reactive reasons, which is consistent with the study by Contreras et al. (2020). Therefore, although some studies have previously analyzed hostile attribution and anger in the context of CPV, our data further delve into the relationship between these variables and this type of family violence, showing its specific relationship with reactive CPV toward both fathers and mothers.

Hypothesis 3 held that a deviant peer group would be positively correlated with drug use, which in turn would be positively linked to CPV motivated by both reactive and instrumental reasons. The analyses confirmed this hypothesis in its entirety both in the case of CPV toward the father and in the case of CPV toward the mother. On the one hand, different studies have revealed a close relationship between a deviant peer group and drug use during adolescence (e.g., Fergusson et al., 2002; Duan et al., 2009; Kendler et al., 2014), and in fact, a deviant peer group predicts drug use in adolescents with CPV (Del Hoyo-Bilbao et al., 2020), so our data agree with these studies. On the other hand, numerous studies have found that drug use is positively associated with violent behaviors of adolescents toward their parents (e.g., Calvete et al., 2011; Ibabe et al., 2013; Beckman et al., 2017; Rico et al., 2017; Rosado et al., 2017; Armstrong et al., 2018).

As mentioned above, the relationship between drug use and the onset of violent behavior is complex. Drug use by adolescents can be a source of conflict between parents and children, and in fact, a significant percentage of adolescents who assault their parents are under the influence of drugs during the aggression (Contreras and Cano-Lozano, 2015). The effect produced by substance use may favor in adolescents the disinhibition that characterizes reactive violence and that, as indicated by Pagani et al. (2009), in confrontations with parents, would increase the likelihood of aggression toward them. Regarding the relationship between drug use and instrumental violence, our results are consistent with previous studies on the subject, which also point to an instrumental use of violence against parents; for example, getting more money from parents is one of the reasons for CPV (Calvete and Orue, 2016; Contreras et al., 2019, 2020). Research on the relationship between a deviant peer group and drug use in the field of CPV has been practically null. Only the work of Del Hoyo-Bilbao et al. (2020) found an indirect effect of the deviant peer group on CPV through drug use, which is in line with our results. In this regard, as suggested by Simmons et al. (2018), it is not clear if peer groups promote CPV behaviors or violence in general or simply support the antisocial lifestyles that adolescents who abuse their parents typically show.

In short, the results of this study confirm the relevant role of various cognitive, emotional, and social variables in the relationship between perceived parental warmth and CPV. Although previous studies have noted the importance of the perceived parental warmth dimension in CPV (Gámez-Guadix et al., 2012; Ibabe et al., 2013; Contreras and Cano-Lozano, 2014; Calvete et al., 2015; Beckman et al., 2017; Zhang et al., 2019), the present study indicates the complexity of this parental dimension in the explanation of CPV and the need to further investigate the mechanisms involved in this relationship.

In conclusion, the lack of perceived parental warmth has important repercussions in the form of psychological maladjustment of children, generating cognitive and emotional problems, which in turn lead to CPV motivated by reactive reasons. Perceived parental criticism-rejection is also correlated with a greater likelihood of association with deviant peer groups, which is associated with drug use and, in turn, with CPV motivated by both reactive and instrumental reasons.

It is necessary to keep in mind the limitations of this study to properly interpret its results. Because it was a cross-sectional study, causal relationships cannot be established between the analyzed variables. The data came from self-reports of the children and therefore refer to the perception they have of their parents. Incorporating joint reports from parents and children would provide us with a more dynamic and complete view of the subject. The relationship between parents and children is interactive and the bidirectional effects cannot be identified in cross-sectional studies. An aggressive adolescent at home causes stress and suffering to parents. In this situation, parents are likely to become more critical and hostile and less warm toward their children. In turn, this can lead to more aggressive behaviors from the adolescent toward their parents, which creates a vicious cycle of family interactions (Gault-Sherman, 2012). Moreover, the data correspond to a sample of Spanish adolescents from the community population, which should be taken into account in the generalization of the data. Future studies could replicate the results with other types of samples. It is also important that future studies analyze the differences between boys and girls in the proposed model as well as to include an analysis of other variables that may mediate or moderate the relationship between parental practices and CPV.

The results of the present study may have important implications in professional practice. Prevention and intervention programs for CPV should consider working with parents on parental practices that incorporate parental warmth as a fundamental element of the psychological adjustment of their children. At the same time, it is important to work with children on dysfunctional aspects of their cognitive and emotional functioning. In turn, it is important to also incorporate into this type of program an analysis of the social context and, more specifically, the possible negative influence of the peer group and of drug use, which can facilitate or intensify violent behaviors toward parents. Although the research on CPV programs is very scare, there are some specific prevention and intervention programs on CPV (e.g., González-Álvarez et al., 2013; Coogan and Lauster, 2015; Ibabe et al., 2018) that include anger control, quality of relationships, drug abuse prevention, etc. Consequently, the findings of the present study are in line with these CPV programs that incorporates the intervention on cognitive, emotional and social variables. Lastly, it is important to keep in mind the different motivations that this type of violence can have. The therapeutic approach depends on whether the violence is reactive in nature or of instrumental use.

## Data Availability Statement

The raw data supporting the conclusion of this article will be made available by the authors, without undue reservation.

## Ethics Statement

The studies involving human participants were reviewed and approved by the Ethics Committee of the University of Jaén (Spain) (Ref. CEIH 270215-1). Written informed consent to participate in this study was provided by the participants’ legal guardian/next of kin.

## Author Contributions

MC-L and LC: conceptualization and methodolgy. SL: validation, formal anaysis, and data curation. MC-L, SL, and LC: writing—original draft preparation; MC-L, FR-D and LC: writing—review, editing and funding adquisition; MC-L: project administration.

## Conflict of Interest

The authors declare that the research was conducted in the absence of any commercial or financial relationships that could be construed as a potential conflict of interest.
